# Towards the Genetic Architecture of Complex Gene Expression Traits: Challenges and Prospects for eQTL Mapping in Humans

**DOI:** 10.3390/genes13020235

**Published:** 2022-01-26

**Authors:** Chaeyoung Lee

**Affiliations:** Department of Bioinformatics and Life Science, Soongsil University, 369 Sangdo-ro, Dongjak-gu, Seoul 06978, Korea; clee@ssu.ac.kr

**Keywords:** complex phenotype, expression quantitative trait locus, regulation of gene expression, statistical genetics, target gene

## Abstract

The discovery of expression quantitative trait loci (eQTLs) and their target genes (eGenes) has not only compensated for the limitations of genome-wide association studies for complex phenotypes but has also provided a basis for predicting gene expression. Efforts have been made to develop analytical methods in statistical genetics, a key discipline in eQTL analysis. In particular, mixed model– and deep learning–based analytical methods have been extremely beneficial in mapping eQTLs and predicting gene expression. Nevertheless, we still face many challenges associated with eQTL discovery. Here, we discuss two key aspects of these challenges: 1, the complexity of eTraits with various factors such as polygenicity and epistasis and 2, the voluminous work required for various types of eQTL profiles. The properties and prospects of statistical methods, including the mixed model method, Bayesian inference, the deep learning method, and the integration method, are presented as future directions for eQTL discovery. This review will help expedite the design and use of efficient methods for eQTL discovery and eTrait prediction.

## 1. Introduction

Despite considerable progress in the 20 years since the completion of the Human Genome Project, a complete understanding of the functions of the human genome sequence, comprising >3 billion base pairs, remains still elusive. Genome-wide association studies (GWASs) have identified thousands of quantitative trait loci (QTLs) as association signals for human complex traits. Because the QTLs have mostly been mapped in the noncoding regions of the human genome, knowledge on their underlying genetic mechanisms are limited. Indeed, candidate causal genes corresponding to QTLs are often falsely assigned because the gene closest to a given QTL is suspected as a target. Studies on expression QTL (eQTL) mapping have aimed to identify their target genes (eGenes) and understand the genetic mechanisms underlying their expression (eTraits) and phenotypic traits. However, these studies are just the tip of the iceberg when considering the eQTL studies required in the future. Herein, human eQTL mapping will be discussed, focusing on challenges and prospects. Detailed theories and equations of methodologies will not be discussed in depth in order to appeal to a wider range of geneticists and fields, including those with practical applications.

## 2. Ground-Breaking Approach for eQTL Discovery and eTrait Prediction

Various approaches have been used in the past decade to develop analytical models and methods for eQTL discovery. In particular, efficient methods have been intensively studied along with advances in technology, from hybridization-based microarray to next-generation sequencing (NGS)–based RNA-seq. Two landmark approaches will be presented herein as examples.

The mixed model method for genetic analysis was originally devised using pedigree information to explain the polygenic effects treated as random effects in the analytical model [1]. Here, the random effects infer that their parameters are assumed to have random variables (for details, see [2]). The use of the model was then extended to the genome-wide identification of loci associated with phenotypic traits and further with eTraits, explaining the random polygenic effects with genome-wide genotypic information instead of pedigree information [2]. In other words, the mixed model method enables us to discover eQTLs, not only via linkage analysis, but also via association analysis. More importantly, it helps rationally and efficiently correct for population structure and elucidate polygenic effects. As a result, it has greatly contributed to accurate eQTL discovery, and avoiding spurious eQTLs, which is considered one of the main concerns in eQTL studies. This method provides a portion of eTrait variance explained by eQTLs and partitions the portion into subportions according to the classified eQTLs; for example, we can separately estimate the portions for cis-acting eQTLs and trans-acting eQTLs. The mixed models can be used to simultaneously analyse multiple eTraits using genome-wide efficient mixed model association (GEMMA) [3]. Using efficient algorithms such as Cholesky decomposition (a method to produce the product of a lower triangular matrix and its transpose, equivalently to a positive-definite matrix) and Gauss–Seidel iteration (a method to iteratively solve linear equations by successive displacement), eTraits can be predicted with a mixed model framework without incurring a heavy computing burden of inverting a huge matrix [4]. In addition, the mixed model has been used in integrative analyses of eTraits and phenotypic traits to prioritize causal genes of phenotypic variation [5].

More recently, deep learning has been garnering increased popularity in predicting gene expression. Deep learning does not have a statistical framework, unlike other methods, thus, it offers great potential at gaining results that are difficult to obtain from other eQTL studies. Moreover, the rapid growth of relevant data will vastly increase the contribution of deep learning to eQTL studies. Deep learning generally refers to supervised or unsupervised learning using advanced artificial neural networks, often known as deep neural networks. Supervised learning represents predicting categorical or continuous variables using a training data set, whereas unsupervised learning represents studying intrinsic patterns and clustering them based on pattern similarity. In this respect, supervised deep learning is a good choice for predicting gene expression. In particular, the large amount of data generated by high-throughput techniques can provide an absolute condition for the use of supervised deep learning. For example, convolutional neural networks are feed-forward deep neural networks in which every unit in a layer is connected to all the units in the previous layer without forming any cycle of the unit connections. Convolutional neural networks have been used to predict gene expression using proximal promoter sequences and distal enhancer sequences obtained by HiChIP [6]. Recurrent neural networks are deep neural networks in which connections between units have a cyclic structure; these networks have been used to predict differences in gene expression between two cell types using histone modification profiles [7].

These approaches have been applied to eQTL studies and have contributed greatly to the discovery of eQTLs for specific genes and offered further insights into general regulation mechanisms of gene expression (Table 1). Often, these approaches are customized to fit specific study objectives. For example, a mixed model incorporating ancestry effects was applied to identify eQTLs in multi-ethnic, or admixed, populations to avoid confounding with the eQTL effect [8]. Deep learning was also applied using a hierarchical Bayesian model with the posterior of parameters for different tasks such as transcription factor binding, chromatin accessibility, and histone marks [9]. This is useful to identify causal variants among candidate nucleotide sequence variants in a strong linkage.

## 3. Complexity of eTraits

**Figure 1:**
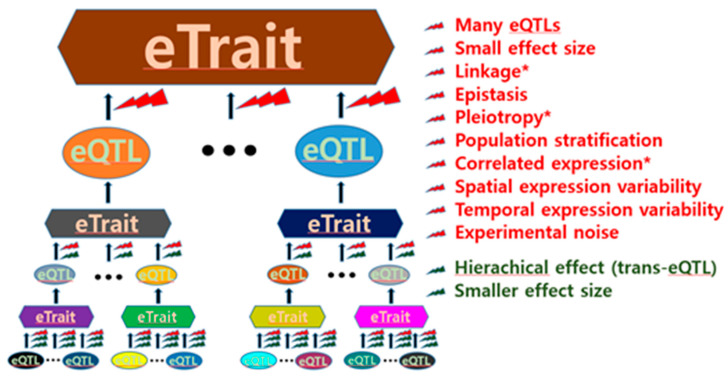
Obstacles to expression quantitative trait locus (eQTL) identification. General obstacles to both cis-eQTL and trans-eQTL identification are presented in red, whereas obstacles to only trans-eQTL identification are presented in dark green. The asterisk indicates obstacles that increase the difficulty of inference of causality.

eQTL discovery is challenging primarily due to its intrinsic complexity. The expression of a single gene in a cell is regulated by various gene products. Each of the various regulating genes in a cell is also regulated by various gene products. This hierarchical regulatory mechanism suggests that many genes expressed in a cell are generally involved in the expression of a single gene (Figure 1), thus having a relatively small effect as an eQTL.

In particular, the effect sizes of trans-eQTLs tend to decrease depending on the degree of trans-acting indirection (Figure 1); therefore, geneticists have experienced difficulties in finding trans-eQTLs. Although the cell environments determined by various conditions are likely genetic, identifying eQTLs can be formidably difficult. Thus, the expressional genetic architecture of many single genes is considerably complex. This was supported by a study where no single eQTL determined mRNA transcription, ribosomal occupancy, or protein abundance of any gene [15]. Hence, the expression of most genes has been referred to as “complex eTraits” hereafter in this article. The complexity of the regulatory function of eQTLs in gene expression can also be attributed to various other factors, as shown in Figure 1. Thus, these factors can act as obstacles to eQTL identification.

Linkages can help researchers find causal sequence variants linked to representative variants associated with gene expression. However, the effect size can be influenced by multiple functional variants in a linkage, often resulting in false positive/negative eQTLs. Many geneticists are therefore reluctant to search for functional sequence variants in the major histocompatibility complex region, where such characteristics resonate extremely well. 

Epistasis among eQTLs is infrequently examined in eQTL mapping because it requires expensive computing power. Epistasis is a natural mechanism that contributes to gene expression regulation by binding DNA and DNA-derived substances (RNA and proteins). For example, eukaryotic transcription is usually initiated by the interplay among transcription factors, activators, mediators, RNA polymerase, enhancers, and proximal or distal promoters. 

Population stratification is also ignored in many eQTL analyses, even though this reduces the accuracy of eQTL mapping and can result in spurious eQTLs [2,20,21]. 

Spatial expression variability ranges from variations among nearby cells to those among organs. In particular, cellular resolution of eQTLs in the brain might be critical for understanding transcriptional heterogeneity of pyramidal cells in addition to regional functions/misfunctions [22,23]. Likewise, temporal expression variability can result from short- to long-term differences, including variability arising from changes in the external environment. 

The aforementioned factors prevent eQTL mapping. In particular, eQTL discovery can be difficult if there are small effect sizes because multiple testing is required to reduce false positives. Inference of causality is also interfered by obstacles such as linkage, pleiotropy, and correlated expression (Figure 1). Further, it is not easy to identify functional nucleotide variants in regions with strong linkages. For example, the human leukocyte antigen (HLA) complex that spans 3.6 megabase pairs covering 224 genes on the short arm of chromosome 6 has many strong linkage disequilibrium (LD) blocks, and the LD blocks are often linked to nucleotide variants outside within the HLA complex [12]. Thus, careful attention is needed to interpret functionality of nucleotide variants, such a complex region in strong linkages.

## 4. Voluminous Work of eQTL Mapping

Owing to the considerable complexity of the eTraits, another critical issue regarding eQTL mapping is the construction of voluminous studies producing a vast amount of data. A genome-wide eQTL analysis produces a genetic profile of regulatory signals for a single gene. In modern studies, eQTL profiles are simultaneously produced for all transcriptome-wide genes. Moreover, integrated analysis of eQTL mapping with GWAS may reveal the regulatory mechanisms underlying phenotypic traits; this is known as a transcriptome-wide association study (TWAS). Profiles can be extended to various types of eQTLs––additive, dominant, recessive, haplotypic, and epistatic. Variations within these profiles can be produced by spatial and temporal gene expression and by various expression molecules across expression processes. Thus, eQTLs are characterized by the expression molecules used for their identification and categorized by expression processes such as transcription and translation (Table 2). The molecular layers can be further extended to DNA or chromatin modification, chromatin interaction, and metabolism. A significant number of profiles beyond those mentioned above are needed in order to understand complexity. For example, eQTL profiles of 13 brain parts are now available from the Common Fund’s Genotype-Tissue Expression Program [24], and more situation-specific single-cell products are becoming available. Profiles of disease states are also necessary to reveal the genetic aetiology; these profiles can be further generated on a population basis, as considered in GWAS for complex diseases. 

## 5. Miscellaneous Issues and Prospects

The issues regarding the qualities and quantities of eQTL maps addressed in this article represent important challenges in statistical genetics. Comprehending the complexities of eTraits is a challenge that transcends the currently available mapping facilities and tools. Recently, there have been substantial improvements in computing environments, which have enabled the analysis of considerable amounts of genomic data, and such developments are constantly accelerating. However, the development of high-resolution eQTL maps requires further marked developments in computing memory and speed. Even now, limitations exist in mixed models that can explain background polygenic effects during eQTL mapping [2]. This is even more problematic when Bayesian inference, which requires intensive computing to marginalize multidimensional joint posterior distribution through the Markov chain Monte Carlo method, is used [16]. Limitations in computing power also render geneticists reluctant to conduct GWASs into the interactions among eQTLs [55]. The computing burden exponentially increases as the interaction order increases. Moreover, while the number of nucleotide variants to be tested increases markedly, interactive variants with low minor allele frequency also likely increase. Identifying higher-order epistasis among eQTLs exhaustively is barely conceivable. Therefore, interactive eQTL mapping relies almost exclusively on experimental studies that may increase the likelihood of interactive functions. For example, a cis-acting eQTL study in which a HiChIP assay for the histone modification of H3K27ac was employed to identify cis-regulatory elements that interacted with the promoters of their target genes, for five types of immune cells, was conducted [32]. More efficient analytical algorithms, methods, and/or designs are required to identify a data-specific workaround, increase the accuracy of eQTL mapping, and decrease the required computing power.

In addition to the abovementioned ones, many other analytical methods have greatly contributed to eQTL mapping for complex eTraits. Nevertheless, the fact that it will likely be impossible to create an ideal eQTL map remains; this is attributed to the great complexity of eQTL maps, as shown in Figure 1. Furthermore, an eQTL map is a moving target that interacts with various environments and is driven by continuous evolution. Innovative progress should be achievable owing to advances in statistical genetics and other disciplines. Future advances in statistical methods for eQTL mapping are highly anticipated, with modifications and extensions of ground-breaking methodologies such as the mixed model, Bayesian inference, deep learning method, and integration method. These provide a critical basis in terms of reducing spurious eQTLs, integrating multiple data, or identifying epistatic eQTLs.

Spurious eQTLs are largely attributed to genetic, environmental, and experimental factors that are ignored or uncounted from the study. Genetic factors include major eQTLs, cis-eQTLs, and trans-eQTLs. A method that can directly explain these genetic factors is the mixed model. As discussed above, the mixed model uses genomic covariance among individuals to explain polygenic effects. The mixed model method reduces residuals unexplained by the analytical model and increases the accuracy of eQTLs [20,56]. It is considered the best statistical tool needed to go one step further to understand the genetic architecture of complex eTraits in the most direct way, without losing any degree of freedom. However, including excessive loci in the construction of genomic covariance or employing the infinitesimal model can lead to spurious eQTLs [56]. The importance of an optimal genomic covariance structure in the mixed model should be stressed. Additional modifications are required in the mixed model for multiple eQTLs with major effects. An example is the multi-locus mixed model analysis in which the analytical model may include additional cofactors by stepwise regression of forward inclusion and backward exclusion [57]. Appropriate adjustment and filtration are required to reduce the errors generated by ignoring environmental and experimental factors. In addition, we need to be wary of eQTLs that can be falsely generated via correlated expression between genes [58]. 

Expensive computing costs to invert a large matrix is a stumbling block in the application of the mixed model method to eQTL mapping, and this block has been encountered in many studies. In particular, because a continuous increase in the sample size in eQTL studies is expected with the development of sequencing technologies, methods for solving or avoiding this problem are required. For example, a reduced animal model equivalent to an animal model has been widely used in genetic analyses for animal breeding; these analyses use a mixed model with pedigree information to reduce the size of the numerator relationship matrix and the number of equations to be solved [59]. Another concern is the violation of the assumption that known variance components are required for the best linear unbiased estimator (BLUE) of the eQTL effect [2]. When we estimate the fixed eQTL effect using polygenic and residual variance component estimates rather than true values, no penalties are imposed, resulting in increased error variability. 

Bayesian inference has several good properties that make it suitable for eQTL mapping. In particular, the Bayesian approach incorporated with a mixed model may overcome the non-BLUE problem raised from the frequentist approach. For example, the Bayesian approach implemented with Gibbs sampling yields polygenic and residual variance components based on polygenic effects and residuals of individuals at every round of the Gibbs chain, finally providing samples of the eQTL effect. Thus, we can directly obtain various point estimates without assuming any distribution. This provides an empirical Bayes estimate of the eQTL effect, corresponding to the BLUE [16]. Nevertheless, mixed model–based Bayesian inference has barely been applied to eQTL analysis. This might be largely attributable to the intensive computing required for the Gibbs chain, resulting in computing costs that would be more expensive than those for the frequentist approach. Hamiltonian Monte Carlo is an efficient Markov chain Monte Carlo method used for the quick convergence of stationary probability distributions; it works by reducing autocorrelation between consecutive samples, which can greatly reduce the computing burden [60]. 

Deep learning is not based on statistical properties; however, it can be used to approach the complexity of eTraits. Deep learning has an advantage in eQTL studies using large amount of data, and it is also a niche approach that is difficult to be used within a statistical framework. However, in the case of deep learning, close attention should be paid to possible issues, such as parameter overfitting, data imbalance, and subtle variances in input data. This is critical to reducing noise and, thus, to unleashing the enormous potential of eQTLs [19]. To apply deep learning, it is also necessary to try to supplement the shortcomings of the difficulty in interpreting the results due to its black box nature.

Various integration algorithms for gene expression analysis have been developed by simultaneously dealing with data from multiple independent studies with comparable designs (horizontal integration) [61] and from multiple molecular measurements on the same subjects (vertical integration) [62]. An integrated analysis combining vertical and horizontal integration is also expected. Another integration analysis helps identify eQTLs simultaneously using multiple tissues, and the analysis may show improved accuracy as well as heterogeneity of eQTLs by tissues. An example is the hierarchical Bayesian model called MT-eQTL used for multiple tissue cis-eQTL analysis [63]. Moreover, further attempts will be made for more diverse types of multidimensional integrated analyses. An example is the TWAS, in which eQTLs and GWAS signals are integrated to identify genes associated with a complex phenotype [64]. TWAS has been extended to the identification of pathways associated with a complex phenotype by aggregating functional annotations across the genes [65]. That is, gene set enrichment analysis can be integrated into gene-based TWAS to generate the pathway-based TWAS. A significant contribution of a pathway to complex phenotypes may be revealed from the set of genes with a small effect size on the pathway [66]. Many extended setups pose challenges because the total number of configurations is likely to grow exponentially, making implementation excessively slow and expensive.

Finally, for researchers wanting to apply analytical methods to eQTL analysis, finding a software with an efficient method suitable for the study purpose is critical to reducing computational costs and appropriately inferencing the results. Analytical methods need to be compared and characterized with various conditions to provide updated and proper guidance, and researchers are desperately in need of a thorough review of analytical methods and software prior to data analysis. For example, if a study on eQTL epistasis is planned, considering the eQTL accuracy and time efficiency, a different optimal method may be chosen according to the number of loci in epistasis and the covariates included in the analytical model. Furthermore, the computing environment should be considered.

This review has primarily focused on analytical methods from the perspective of statistical genetics. Along with the improvement of methods, increasing sample size and advancing sequencing technology are essential to accelerate the development of eQTL studies. Strategies that prioritize increasing sample size may include establishing standards for data and expanding shareable data repositories. Sequencing techniques such as Hi-C, Capture-C, and 3C-seq may improve eQTL studies for chromatin interactions [67]. Finally, advances in fundamental technologies that can be used more broadly could further change the paradigm of the field, as previously shown by microarrays and next generation sequencing.

## 6. Conclusions

Statistical genetics is a key discipline in mapping eQTLs and predicting eTraits. Now, it is time to accelerate the development of analytical methods for solving problems or mitigating limitations. To date, eQTL studies have been strongly biased toward the discovery of cis-eQTLs. This is largely attributable to the easy biological interpretation on direct regulation, the relatively large effect size, and the reduced number of variants to be tested within a certain region. However, we should also make efforts toward developing efficient methods to overcome the limitations of trans-eQTL discovery. Thirty-seven percent of GWAS signals (*p* ≤ 5 × 10^−8^) for the phenotypic traits corresponded to trans-eQTLs that were recently reported using blood samples of 31,684 individuals by the eQTLGen consortium [68]. This proportion will increase with the trans-eQTLs of other tissues and with a larger sample size. Comparing this large-scale analysis to the second-largest analysis for blood eQTLs, where 8% of the GWAS signals were found to be trans-eQTLs using blood samples of 5311 individuals [69], a considerable advantage of larger sample sizes is expected. As great amounts of data are accumulated in the future, the concerns regarding trans-eQTL discovery will reduce. The trans-eQTL profile may reflect a characteristic of tissue-specific regulation that differentiates body parts and developing efficient analytical methods can increase the accuracy.

In conclusion, the genetic architecture of complex eTraits, produced at a higher resolution through a more rational analysis of enormous amounts of gene expression data, will contribute to the understanding of the genetic architecture of complex diseases; this will constitute an important basis in precision medicine.

## Figures and Tables

**Table 1:** Examples of eQTL studies and reviews using a mixed model or a deep learning.

eQTL	Methods	Results	Reference
**Mixed model**			
cis-neQTL	GEMMA	Inflammation-dependent cis-neQTLs in intestine	[10]
neQTL	GaLA-QTLM	neQTL mapping with ancestry data in multi-ethnic population	[8]
neQTL	MM with pedigree-based covariance matrix	Cell type-specific neQTLs in brain and blood for Alzheimer’s disease	[11]
neQTL	BOLT-LMM, GEMMA	Cis-/trans-neQTLs in peripheral blood and their contribution to heritability	[12]
cis-neQTL	StructLMM	Cell-context interaction with environmental variables	[13]
neQTL, rQTL, pQTL	AIREML, BLUP	Efficient translational control of pQTLs for ribosomal protein genes	[14]
neQTL, rQTL, pQTL	AIREML	Inclusion of optimal number of eQTLs in constructing polygenic covariance matrix	[15]
General	REML and others	Review on frequentist mixed model methodology	[2]
General	Gibbs sampling, MCMC	Review on Bayesian mixed model methodology	[16]
**Deep learning**			
meQTL	CpGenie (CNN)	Prediction of the allele-specific impact of nucleotide variants on proximal CpG methylation	[17]
cQTL	DeepHiC (CNN)	Functional prediction of nucleotide substitution on chromatin interaction using Hi-C data	[18]
neQTL, dsQTL, atacQTL	MtBNN (CNN, RNN)	Incorporating a Bayesian approach to assessing functional impact of non-coding variants	[9]
General	DNN	Introduction to deep learning for genomics covering more than eQTL mapping	[19]

neQTL, narrow-sense expression quantitative trait locus; rQTL, ribosome occupancy QTL; pQTL, protein abundance QTL; meQTL, methylation QTL; cQTL, chromatin interaction QTL; dsQTL, DNase sensitivity QTL; atacQTL, assay for transposase accessible chromatin QTL; GEMMA, genome-wide efficient mixed-model association; MM, mixed model; AIREML, average information restricted maximum likelihood; BLUP, best linear unbiased prediction; MCMC, Markov chain Monte Carlo; CNN, convolutional. neural network; RNN, recurrent neural network; DNN, deep neural network.

**Table 2:** Types of expression quantitative trait loci (eQTLs) associated with various molecular layers.

Name	Abbrev.	Molecular Phenotype Associated with eQTL (Method)	Ref.
**Chromatin modification**			
chromatin accessibility QTL	caQTL	Active and potential regulatory DNA elements, e.g., dsQTL (DNase-seq, ATAC-seq)	[25,26,27]
methylation QTL	meQTL	DNA methylation for altering chromatin structure, mainly in regulatory regions such as promoters and intron–exon boundaries (ChIP-seq)	[28]
histone QTL	hQTL	Magnitude of histone post-translational modifications for chromosomal packaging, e.g., H3K4me3 for promoters, H3K4me1 for enhancers, H3K27ac for promoters and enhancers (ChIP-seq)	[27,29,30]
TF binding QTL	bQTL	Transcription factor binding, e.g., NF-κB, PU.1/Spi1, Stat1, JunD, and Pou2f1/Oct1 (ChIP-seq)	[30,31]
**Chromatin interaction**			
promoter interacting eQTL	pieQTL	eQTLs overlapping active cis-regulatory elements that interact with their target gene promoters (HiChIP)	[32]
chromatin interaction QTL	cQTL	Allelic differences of chromatin interactions between two homologous chromosomes mediated by CTCF and RNAPII (ChIA-PET)	[33]
promoter enhancer interaction QTL	peQTL	Allele-specific RNAPII-mediated chromatin interactions with phased transcript (ChIA-PET)	[33]
**Transcription**			
narrow-sense eQTL	neQTL	Gene expression level as the sum of all transcripts of each gene. We used the “neQTL” to differentiate this from eQTL, a generic term for all kinds (RNA-seq)	[34,35]
miRNA eQTL	miR-eQTL	Expression level of miRNA for post-transcriptional and translational regulation (small RNA-seq)	[34,36]
lncRNA eQTL	lncR-eQTL	Expression level of lncRNA for transcriptional, post-transcriptional, and epigenetic regulation (RNA-seq)	[34,37,38]
circRNA-eQTL	Circ-eQTL	Expression level of circRNA for sequestration of miRNAs/proteins, splicing interference, and transcriptional and translational regulation (RNA-seq)	[39,40]
response eQTL	reQTL	Transcriptomic response to external stimuli (RNA-seq)	[41,42]
repeat eQTL	repeat-eQTL	Retrotransposon-derived repeat element as a source for evolution of new transcripts (RNA-seq)	[34]
splicing QTL	sQTL	Relative abundance of the transcript isoforms of a gene or the intron excision ratios of an intron cluster for regulation of alternative splicing (RNA-seq)	[34,43]
transcript ratio QTL	trQTL	Ratio of each transcript to the total gene expression for transcript usage, splicing, and transcript structure (RNA-seq)	[34]
Allele specific expression QTL	aseQTL	Transcription differences between two different haplotypes in a heterozygous individual (RNA-seq)	[34]
poly(A) ratio QTL	apaQTL	Alternative polyadenylation for mRNA stability and translation efficiency (RNA-seq)	[44]
RNA editing QTL	edQTL	RNA editing level for post-transcriptional processes such as RNA splicing, localization, stability, and translational efficiency (RNA-seq)	[45]
m^6^A QTL	m^6^A-QTL	N^6^-methyladenosine level in mRNA transcript for mRNA processing. (m^6^A-seq)	[46]
RNA synthesis rate QTL	rsQTL	Transcription rates (4sU-seq)	[47]
RNA decay QTL	rdQTL	mRNA decay rates for modulating steady-state transcript levels (RNA-seq)	[48]
transcription initiation QTL	tiQTL	Activity of transcribed transcriptional regulatory elements (tTREs) in promoter and enhancer region (PRO-seq)	[49]
directional initiation QTL	diQTL	Directionality of divergent bidirectional transcription at tTREs using log ratio of plus strand reads over minus-strand reads (PRO-seq)	[49]
**Translation**			
ribosome occupancy QTL	rQTL	Ribosome occupancy for translational regulation and translation efficiency (Ribo-seq)	[50]
protein abundance QTL	pQTL	Protein expression level for post-transcriptional regulation (mass spectrometry)	[50]
**Metabolism**			
Metabolite QTL	mQTL	Small endogenous molecules or metabolites that reflects the dynamic response to physiological, pathophysiological, and/or developmental stimuli (NMR or mass spectroscopy)	[51,52]
microbiome QTL	mbQTL	Microbial composition in multiple host tissues such as gut and skin (16S rRNA and ITS sequencing)	[53,54]

## Data Availability

Not applicable.
